# Supplementary material for: A haplotype-complete chromosome-level assembly of octoploid Urochloa humidicola cv. Tully reveals multiple genomic compositions and evolutionary histories in the species
Source: G3 (Bethesda). 2026 Feb 12;16(4):jkag033. doi: 10.1093/g3journal/jkag033 (PMC13042314; doi:10.1093/g3journal/jkag033)

Hap1 vs p_ctgs

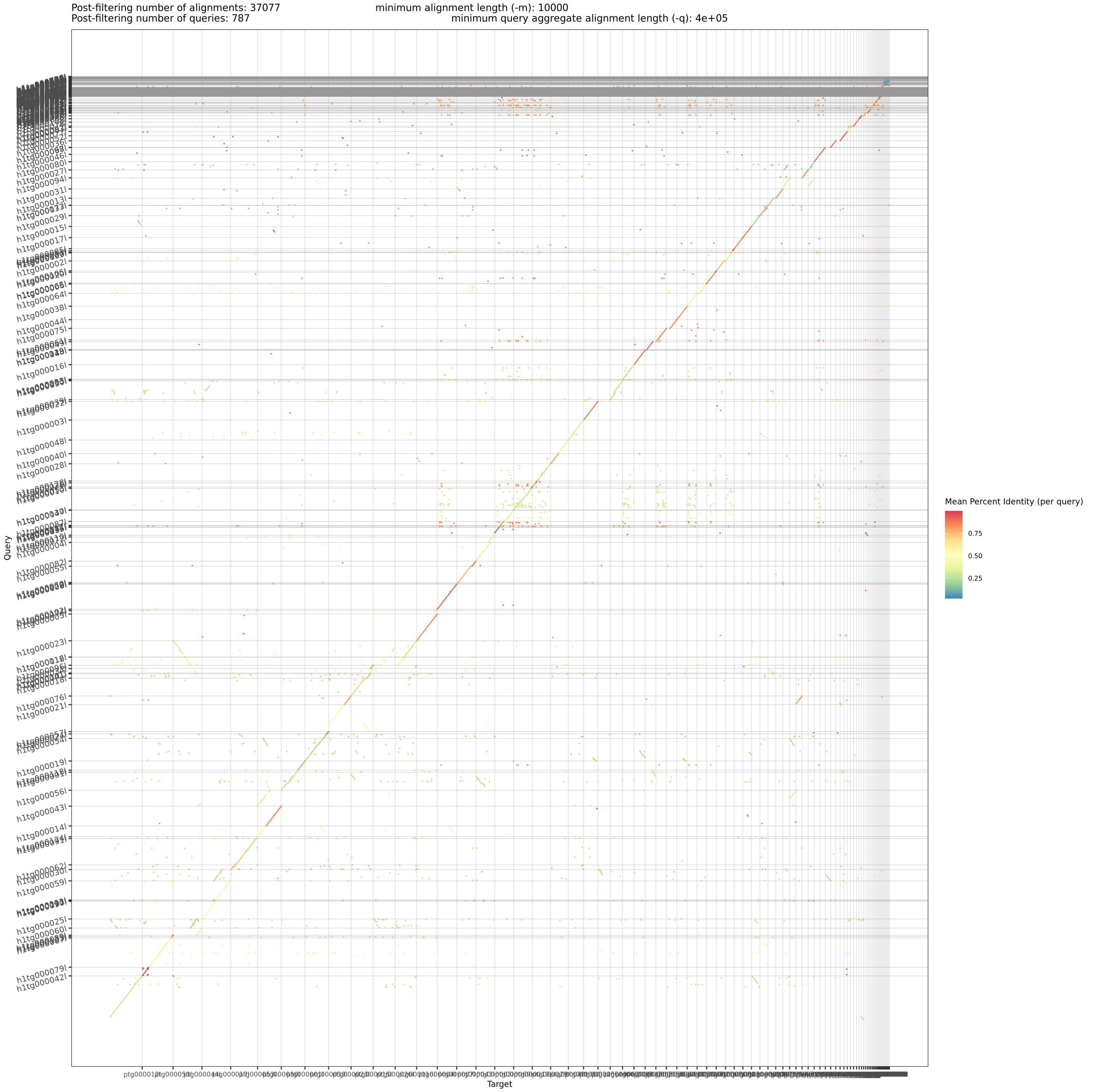

Hap2 vs p_ctgs

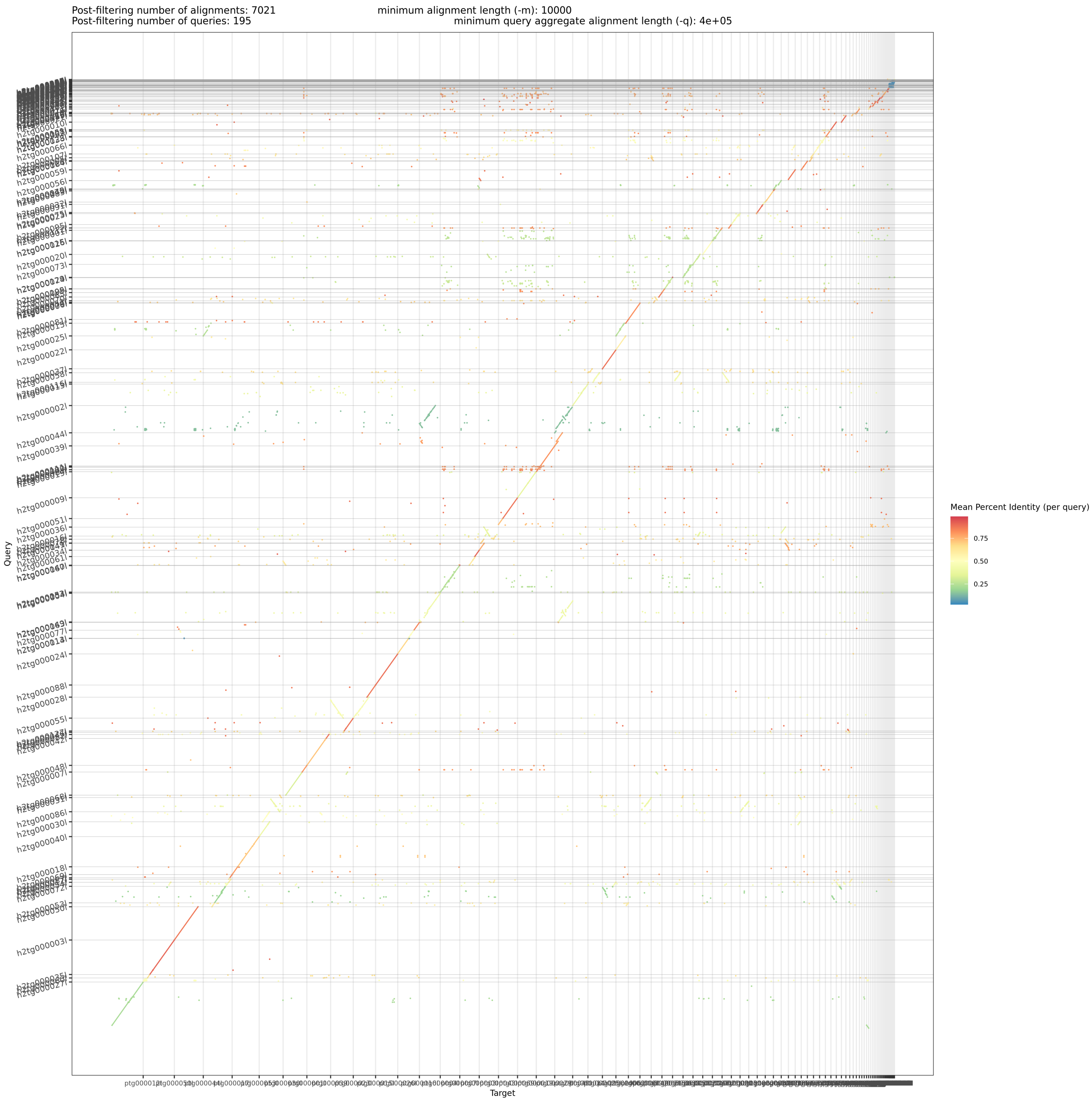

Hap1 vs Hap2

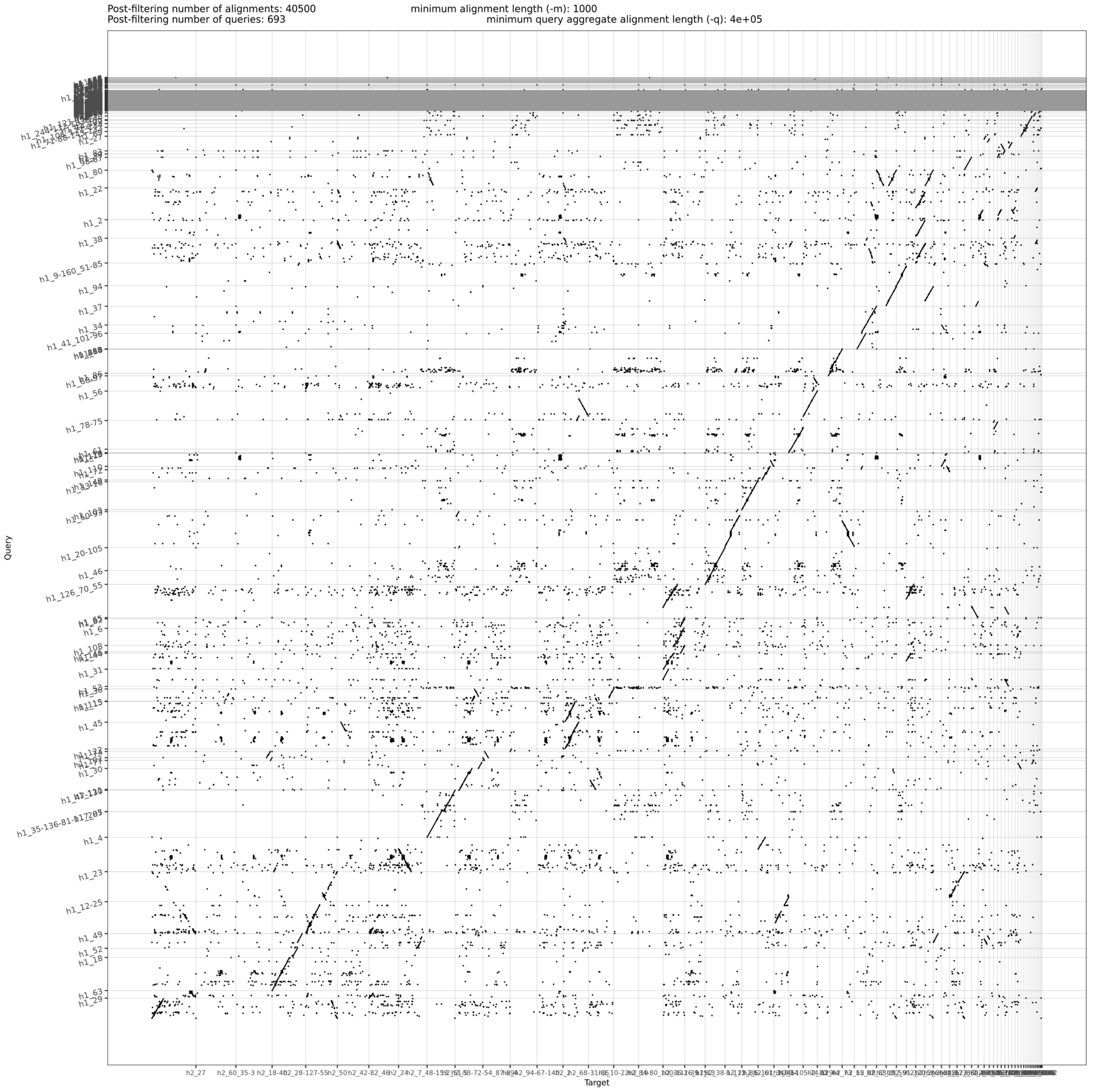
Post-filtering number of alignments: 40500
Post-filtering number of queries: 693
minimum alignment length (-m): 1000
minimum query aggregate alignment length (-q): 4e+05
Query
Target
h1_27
h1_80
h1_22
h1_2
h1_38
h1_9-160_51-85
h1_94
h1_37
h1_34
h1_41_101-96
h1_86
h1_56
h1_78-75
h1_61
h1_110
h1_20-105
h1_46
h1_126_70_55
h1_6
h1_108
h1_31
h1_45
h1_30
h1_4
h1_23
h1_12-25
h1_49
h1_52
h1_18
h1_63
h1_29
h2_27
h2_60_35-3
h2_50
h2_24

Hap2 vs Hap1

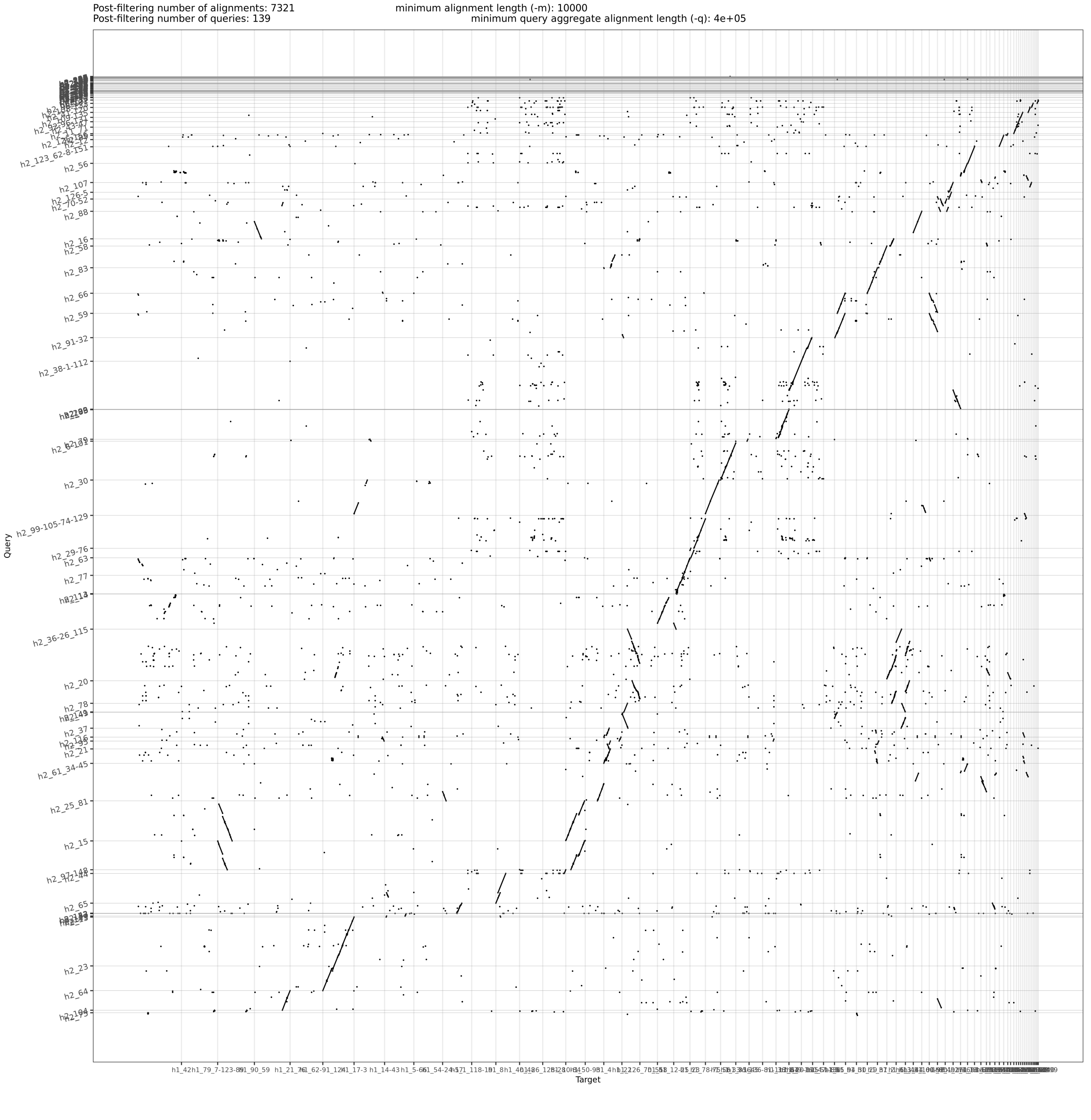

Post-filtering number of alignments: 7321
Post-filtering number of queries: 139
minimum alignment length (-m): 10000
minimum query aggregate alignment length (-q): 4e+05
Query
Target
h2_123_62-8-151
h2_56
h2_107
h2_126-5
h2_70-52
h2_88
h2_16
h2_58
h2_83
h2_66
h2_59
h2_91-32
h2_38-1-112
h2_30
h2_99-105-74-129
h2_29-76
h2_63
h2_77
h2_36-26_115
h2_20
h2_78
h2_37
h2_21
h2_61_34-45
h2_25_81
h2_15
h2_97-148
h2_65
h2_23
h2_64
h1_42
h1_90_59
h1_17-3
h1_14-43

Hap1 vs U fusca v1.0

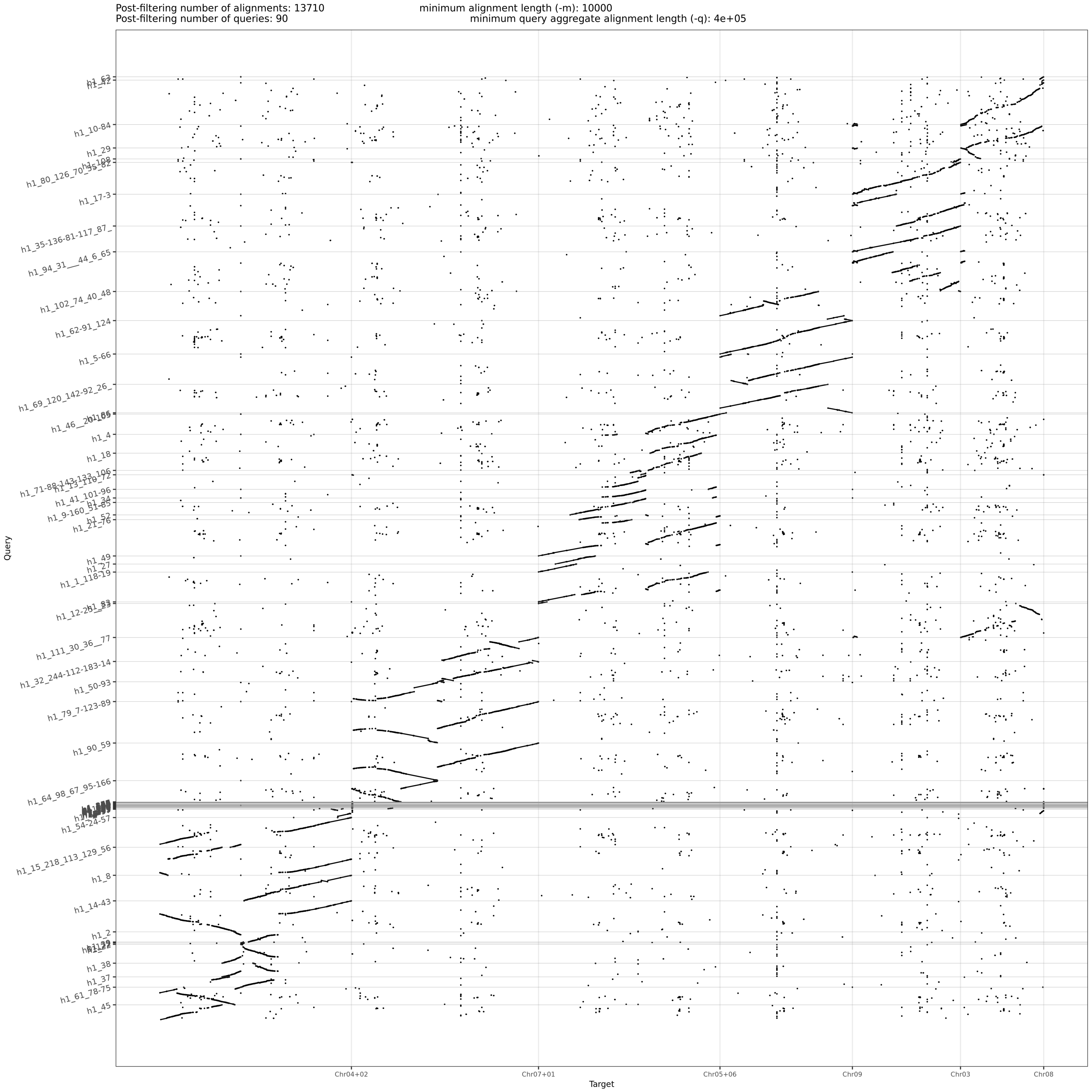

Hap2 vs U fusca v1.0

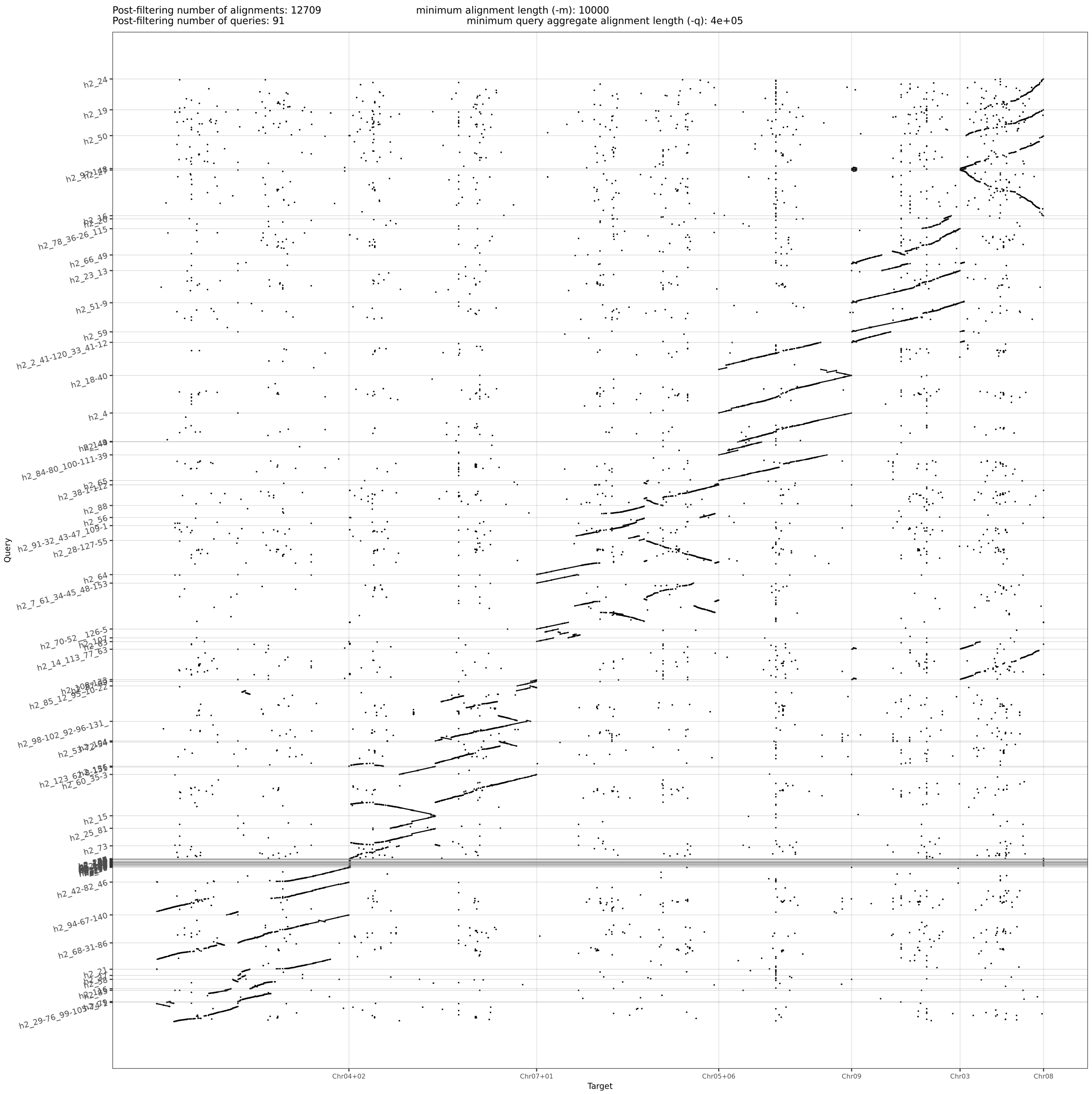

Hap1+hap2 vs U fusca v1.0 (first round)

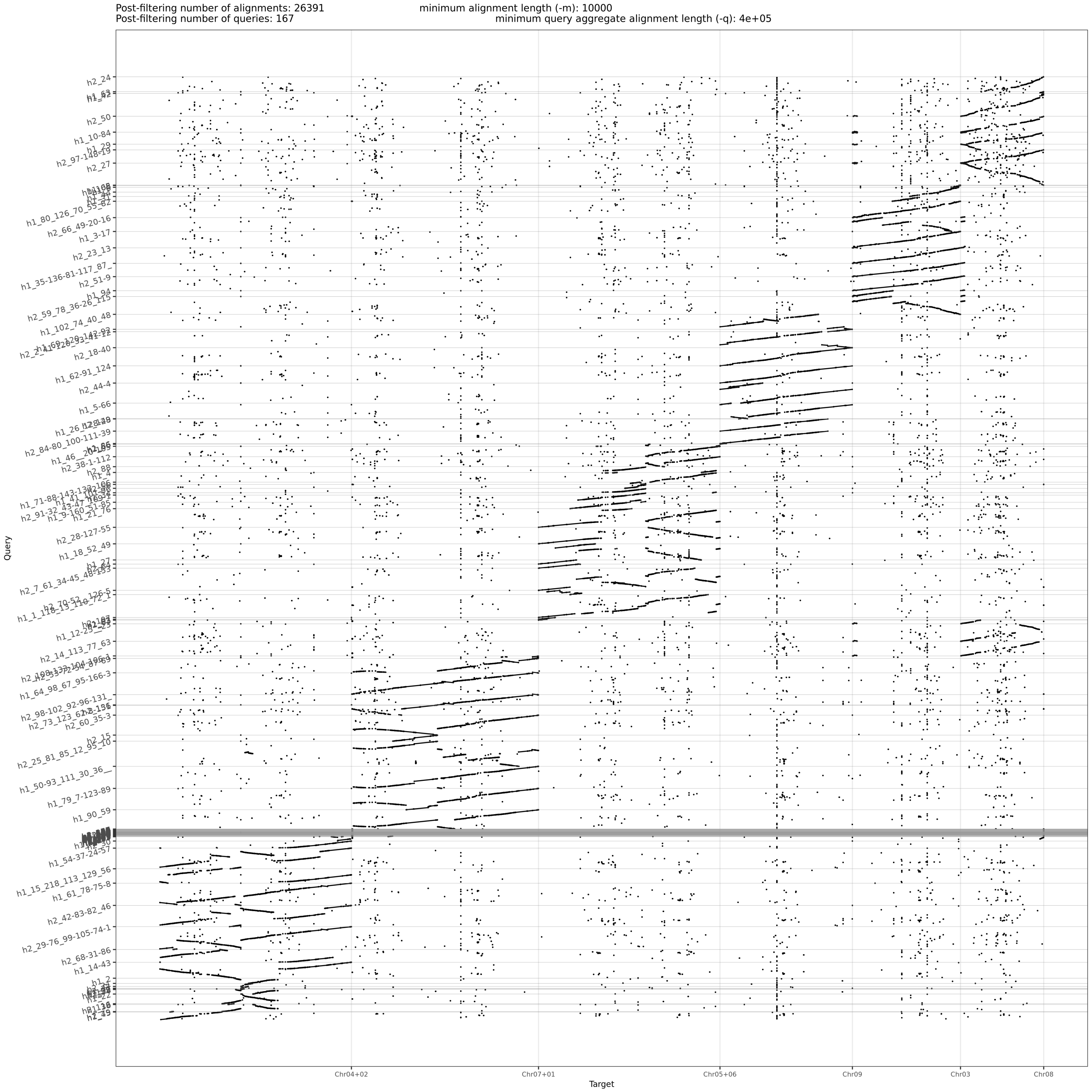

Hap1+hap2 vs U fusca v1.0 (second round)

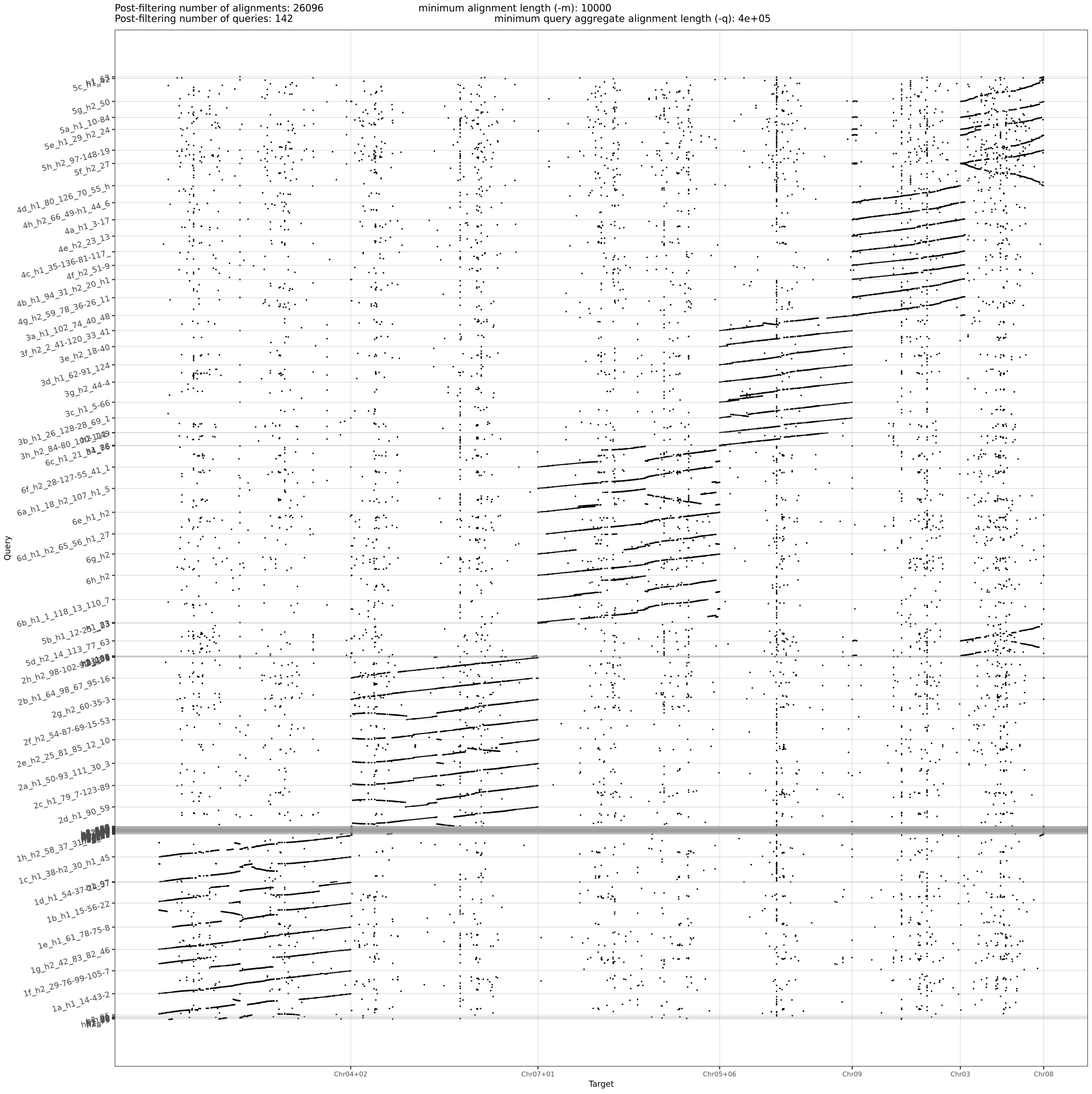

Supplement: jkag033_Supplementary_Data [file jkag033_supplementary_data.zip › Supplementary_file_1_G3-2025-406458.pdf]
